# Mechanism-Based Cardiac Regeneration Strategies in Mammals

**DOI:** 10.3389/fcell.2021.747842

**Published:** 2021-10-11

**Authors:** Nawazish Naqvi, Siiri E. Iismaa, Robert M. Graham, Ahsan Husain

**Affiliations:** ^1^Division of Cardiology, Department of Medicine, Emory University School of Medicine, Atlanta, GA, United States; ^2^Victor Chang Cardiac Research Institute, Darlinghurst, NSW, Australia

**Keywords:** heart failure, ischemic injury, cardiomyocyte proliferation, cardioprotection, cardiac regeneration

## Abstract

Heart failure in adults is a leading cause of morbidity and mortality worldwide. It can arise from a variety of diseases, with most resulting in a loss of cardiomyocytes that cannot be replaced due to their inability to replicate, as well as to a lack of resident cardiomyocyte progenitor cells in the adult heart. Identifying and exploiting mechanisms underlying loss of developmental cardiomyocyte replicative capacity has proved to be useful in developing therapeutics to effect adult cardiac regeneration. Of course, effective regeneration of myocardium after injury requires not just expansion of cardiomyocytes, but also neovascularization to allow appropriate perfusion and resolution of injury-induced inflammation and interstitial fibrosis, but also reversal of adverse left ventricular remodeling. In addition to overcoming these challenges, a regenerative therapy needs to be safe and easily translatable. Failure to address these critical issues will delay the translation of regenerative approaches. This review critically analyzes current regenerative approaches while also providing a framework for future experimental studies aimed at enhancing success in regenerating the injured heart.

## Introduction

About seven million Americans are currently living with heart failure; a number expected to increase to ∼8.5 million by 2030 (Urbich et al., 2020). The total healthcare cost for heart failure was ∼$43 billion in 2020 and, in the absence of a breakthrough in therapy that significantly improves outcomes, is expected to increase to ∼$70 billion by over the next decade (Urbich et al., 2020). This situation is further compounded by an aging population. Treatment for heart failure generally consists of afterload reduction with one or more of several different classes of antihypertensive drugs, diuretics, or both, which generally need to be continued long-term. Despite this therapy, ∼50% of the patients who develop heart failure die within 5 years of diagnosis (Taylor et al., 2017; Jones et al., 2019), a death rate higher than some cancers. In addition, heart failure is associated with a marked deterioration in quality-of-life as it not infrequently leads to depression, hostility and impaired physical activity (Dracup et al., 1992; Bennet et al., 2002; Hobbs et al., 2002; Cai et al., 2019). At present the only definitive therapy for severe heart failure is heart transplantation. However, this requires major expensive surgery and life-long medical management including immunosuppressive therapy with attendant risks of infection and the development of malignancies. Moreover, the number of patients eligible for a heart transplant far exceeds donor organ availability.

Thus, heart failure represents a huge unmet need for the development of next generation therapies that can definitively address the loss of cardiomyocytes associated with ischemic and many other types of heart disease that result in impaired contractile function. Cardiac regeneration as a strategy for reversing heart failure offers promise because it will allow the myocardium to be rebuilt with attendant improvements in left ventricular (LV) contractility and reversal of adverse LV remodeling.

Initial (first generation) approaches to induce cardiac regeneration focused mainly on cell-based therapies, such as bone marrow derived stem cells or resident cardiac stem cells (Orlic et al., 2001; Chugh et al., 2012; Ellison et al., 2013). These studies assumed that exogenous cells delivered into the heart would engraft and transdifferentiate into functional cardiomyocytes, or that endogenous cardiac stem cells would mature and functionally couple with existing cardiomyocytes. It is now clear that success with these approaches has been limited because engraftment and survival of exogenous stem cells is poor, so benefits are likely largely restricted to short term paracrine effects or immune response triggered by the injected cells (Vagnozzi et al., 2020), rather than conversion into functional, electrically coupled cardiomyocytes. In addition, the adult heart contains few if any endogenous stem cells that can differentiate into cardiomyocytes (van Berlo et al., 2014; Kanisicak et al., 2017).

Another approach to cardiomyocyte regeneration involved differentiation of embryonic stem cells (ESCs) and induced pluripotent stem cells (iPSCs) to cardiomyocytes *in vitro* and then injecting these pluripotent stem cell-derived cardiomyocytes (ESC-CMs or iPSC-CMs) directly into the heart after an ischemic injury (Caspi et al., 2007; Fernandes et al., 2010). Studies by Murry and co-investigators showed the feasibility of producing human ESC-CMs in large scale with good viability; a requisite first step in the potential translation of this technology (Chong et al., 2014). In this study, intramyocardial injection of ∼1 billion ESC-CMs into the infarct border zone 2 weeks after myocardial ischemia–reperfusion (IR) injury in pigtail macaque monkeys resulted in reduced infarct size—these ESC-CMs showed progressive but incomplete maturation over the following 3 months. Despite retention of these ESC-CMs, LV contractile function did not improve. Importantly, Chong et al. (2014) reported arrhythmias in monkeys injected with ESC-CMs, which may be related to imperfect electrical coupling to preexisting cardiomyocytes. Thus, arrhythmogenesis and the need for long-term immunosuppression continue to present significant hurdles for the clinical application of this regenerative approach (Laflamme and Murry, 2011). Although, this field has progressed considerably from these initial studies, significant challenges and limitations of the technique remain (this topic has recently been reviewed by Gao and Pu, 2021).

An alternate approach to cardiac regeneration is to induce gene transcription programs *in situ* in non-myocytes to convert them into induced cardiac-like myocytes (iCLMs). Because cardiac fibroblasts are abundant in the heart and can be reprogrammed after activation in response to injurious stimuli such as ischemia, they have been targeted to form iCLMs. To this end, intramyocardial injection of cardiac fibroblasts in mice, one day after their transduction with three transcription factors (*Gata4*, *Mef2c* and *Tbx5*) *in vitro*, showed reprogramming into iCLMs *in vivo* (Ieda et al., 2010). In mice, intramyocardial injection of retroviral vectors encoding four transcription factors (*Gata4*, *Hand2*, *Mef2c* and *Tbx5* designated as GHMT) also resulted in iCLMs *in vivo* (Song et al., 2012). However, additional factors were required for reprogramming human cells (see Yamakawa and Ieda, 2021 for a detailed review). Importantly, in mice, this therapy improved LV contractility (ejection fraction (EF) at 24 h post-myocardial infarction (post-MI) and reduced subsequent infarct extension (Song et al., 2012). Given that GHMT was administered immediately after MI injury and the efficiency of fibroblast reprogramming to iCLMs is quite low, it is unclear if improvements in contractile function and scar size were due to iCLM formation or to paracrine effects of the transduced fibroblasts. Such effects could include reduced death of endogenous cardiomyocytes, decreased replacement fibrosis, modulation of profibrotic and scar-maturing immune cells or myofibroblasts numbers. While this approach is innovative, the low efficiency of reprogramming vectors remains a challenge (Sadahiro et al., 2015). The need to directly inject reprogramming factors into the myocardium is also a significant hurdle for the clinical application of this approach.

**FIGURE 1:**
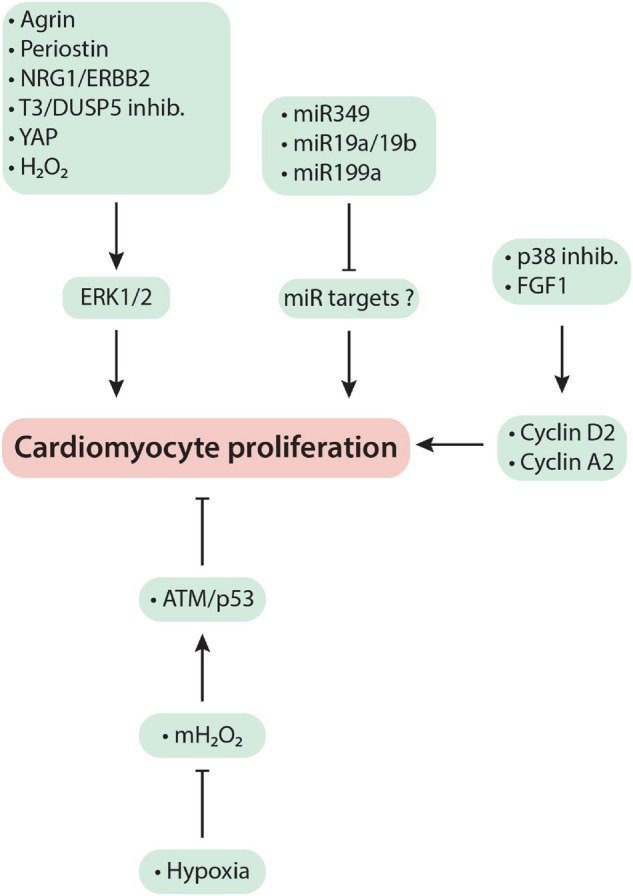
Mechanism-based cardiac regeneration strategies in mammals. See text for a detailed discussion of the mechanisms exploited by each regenerative therapy.

Mechanism-based regenerative therapies have been developed by studying when and how cardiomyocytes lose their ability to proliferate after birth with the hope of identifying molecular targets that could be manipulated to regain adult cardiomyocyte proliferation in order to regenerate cardiac muscle that has been lost due to a myocardial injury. Diverse signaling pathways are involved, but as Figure 1 and the discussion below show, mitogenic signaling by ERK1/2 (extracellular signal-regulated protein kinase1/2) is central to most of the regenerative therapies that demonstrate functional benefit in preclinical research. The focus of this review is on such mechanism-based approaches with an emphasis on their therapeutic potential in regenerating the adult heart after myocardial injury.

## Loss of Proliferative Potential in Postnatal Hearts

An early study suggested that cardiomyocytes in the murine heart cease to divide from birth onwards (Soonpaa et al., 1996). However, many other studies have shown substantial cardiomyocyte proliferation during the early neonatal period (Li et al., 1996; Naqvi et al., 2014; Alkass et al., 2015). The finding that markedly injured postnatal day (P)1 (Porrello et al., 2011) or P2 (Naqvi et al., 2014) murine hearts undergo complete regenerative repair, by cardiomyocyte replication over the subsequent 21 days, indicates that loss of cardiomyocyte replicative potential in mice cannot occur at or before birth.

The finding that efficient post-injury cardiac regeneration, in mice, is lost after P7 could mean that cardiomyocytes lose proliferative capacity immediately after the end of the neonatal period, as suggested by Porrello et al. (2011). This view has support from the findings of Alkass et al. (2015) and Soonpaa et al. (2015) which show no evidence of cardiomyocyte number expansion or cardiomyocyte S-phase entry, respectively, after P7. However, quantitation of cardiomyocyte numbers in healthy hearts of multiple species has questioned this conclusion. For example: Mollova et al. (2013) reported a 3.4-fold increase in cardiomyocyte numbers between the first year and 20 years of life in humans; Chen et al. (2007) reported a 12% increase during early adolescence in feline hearts; and Naqvi et al. (2014) and Puente et al. (2014) reported ∼40% (P10→P18) and ∼30% (P7→P21) increases, respectively, during preadolescence in murine hearts. This level of cardiomyocyte number expansion suggests that substantial number of cardiomyocytes should be entering S-phase and mitosis during preadolescence. As a corollary to Naqvi et al. (2014), a blinded analysis of preadolescent heart sections by Harvey’s laboratory confirmed extensive cardiomyocyte mitosis using aurora B labeling (Naqvi et al., 2015). For mitosis to occur, S-phase must have successfully completed. However, Soonpaa et al. (2015) using BrdU labeling could not find cardiomyocytes entering S-phase during preadolescence. By contrast, findings from Shah and Brewer’s laboratories indicated that a single injection of BrdU given to mice on P14 labeled 2.8% of cardiomyocytes over the following 16 h (Murray et al., 2015). Naqvi et al. (2014) also reported an ∼10% labeling of cardiomyocytes following a BrdU pulse in P14 mice. While these percentages are less than the level of cardiomyocyte number expansion observed during preadolescence/early adolescence in mice, cat and humans (Chen et al., 2007; Mollova et al., 2013; Naqvi et al., 2014; Puente et al., 2014), it is important to note that the ∼2 h half-life of BrdU in circulation (Kriss and Revesz, 1962) is unlikely to label all cardiomyocytes that enter the cell cycle during this developmental period.

Methodological differences between laboratories in estimating cardiomyocyte proliferation could be a source of these conflicting reports as discussed in detail below in “ASSESSMENT OF EXTANT PRECLINICAL CARDIAC REGENERATION RESEARCH.” Alternatively, these divergent findings may also be reconciled if ventricular cardiomyocytes in the immediate post-neonatal period are heterogeneous in relation to their proliferative potential, with a sub-population retaining responsiveness to mitogenic stimuli. Bogush et al. (2020) tested this hypothesis and, using genetic lineage tracing, showed that P8 cardiomyocytes of the LV apex proliferate in response to a mitogenic stimulus, but those of the LV base do not (Bogush et al., 2020). After P16, LV cardiomyocytes uniformly become mitotically quiescent. Thus, spatiotemporal heterogeneity in cardiomyocyte proliferative capacity, coupled with methodological differences between laboratories, might explain the divergent findings between laboratories that have evaluated post-neonatal cardiomyocyte proliferative potential (Naqvi et al., 2014; Alkass et al., 2015; Naqvi et al., 2015; Soonpaa et al., 2015; Hirai et al., 2016).

### Binucleation and Increased Ploidy as a Gauge of Proliferative Incompetence in Cardiomyocytes

Cardiomyocyte cell cycle entry may not always result in cell division. If progression through the cell cycle is aborted after S-phase but before karyokinesis (nuclear division), each cardiomyocyte nucleus ends up with twice the genetic material (*4c*, or higher, DNA content than normal diploid *2c* DNA content). This phenomenon may be defined as nuclear polyploidy. If cell cycle progression is aborted after karyokinesis, but before cytokinesis (cell division), the cell ends up with two nuclei (a binuclear cardiomyocyte). In mice, cardiomyocyte nuclear polyploidy (≥ *4n*) (Patterson et al., 2017) and binucleation (Soonpaa et al., 1996) increase during the fetal to neonatal transition. Several investigators have noted an association between a diminished regenerative potential of a heart and the presence of nuclear polyploidy and/or multinucleation of its constituent cardiomyocytes and have concluded that cardiomyocytes with increased nuclear ploidy or those that have multiple nuclei are terminally differentiated (Patterson et al., 2017); that is, these cells have permanently exited the cell cycle (Derks and Bergmann, 2020).

Increased nuclear polyploidy or multinucleation are indicators of stalled cell cycle progression. But, are these cells incapable of subsequent proliferation? In the liver, binucleated tetraploid hepatocytes arise due to a failure in cytokinesis (Celton-Morizur et al., 2009). However, after hepatic injury they rapidly divide to effect complete liver regeneration (Miyaoka et al., 2012).

*In vitro* studies indicate that binuclear cardiomyocytes are capable of proliferation. Engel et al. (2005), D’Uva et al. (2015), and Wang et al. (2017) reported several examples and modes of division of large adult binuclear murine cardiomyocytes. However, it is unknown if mitogen-induced cardiomyocyte proliferation *in vivo* predominantly stimulates cytokinesis of mononuclear or binuclear cardiomyocytes. This may depend not only on the stimulus threshold that must be overcome to stimulate proliferation of mono- or binuclear cardiomyocytes, but also the percentage of mono- and binuclear cardiomyocytes that populate individual hearts. These issues may be important in the translation of regenerative therapies to humans because unlike mice, human cardiomyocytes are predominantly mononuclear but polyploid (Botting et al., 2012; Derks and Bergmann, 2020).

### Role of Early Developmental Shift in Metabolism in Inducing Cardiomyocyte Cell Cycle Arrest

Sadek and co-investigators (Puente et al., 2014) proposed that anaerobic glycolysis is the primary source of energy for mammalian cardiomyocytes in the hypoxic fetal environment and that, after birth, a switch from hypoxia to normoxia triggers a change from glycolysis to mitochondrial oxidative phosphorylation (OXPHOS) as the energy source. Mitochondrial OXPHOS generates hydrogen peroxide (mH_2_O_2_) as a byproduct of OXPHOS reactions. If the levels become sufficiently high, mH_2_O_2_ could activate DNA damage response (DDR) signaling and result in cell cycle arrest in murine cardiomyocytes after P6 (Puente et al., 2014). This hypothesis predicts that, after P6, cardiomyocytes are perpetually subjected to heightened DDR signaling. A key DDR effector is the tumor suppressor protein p53, actions of which include cell-cycle arrest and apoptosis. Teleologically, heightened DDR signaling in adult cardiomyocytes is inconsistent with the extraordinarily long life of mammalian cardiomyocytes—in humans, some of these cells survive for more than half a century.

A major controversy surrounds the hypothesis presented by Puente et al. (2014). We briefly present some of the opposing studies. Triiodothyronine (T3; a thyroid hormone) is the key regulator of mitochondrial biosynthesis and OXPHOS in mammalian cardiomyocytes (Turrens, 2003; Marín-García, 2010; Li et al., 2014). Hirose et al. (2019) proposed that, during the fetal to neonatal transition, a developmental increase in circulating T3 at ∼P4, rather than normoxia, induces murine cardiomyocyte cell cycle arrest because T3, by enhancing mitochondrial OXPHOS, would be primarily responsible for increases in mH_2_O_2_ and DDR signaling. These ideas have been explored by others through studies on the *in vivo* actions of both H_2_O_2_ and T3. Overexpression of the H_2_O_2_-generating enzyme NADPH oxidase 4 (Nox4) in murine cardiomyocytes demonstrated that, in P14 hearts, H_2_O_2_-activates ERK1/2 signaling, which results in c-myc-mediated cyclin D2 expression and BrdU incorporation in cardiomyocytes (Murray et al., 2015). In neonatal mice, exogenous T3 increases *in vivo* cardiomyocyte proliferation without nuclear polyploidization or multinucleation (Tan et al., 2019). Overexpression of mitochondria-localized catalase, which scavenges mH_2_O_2_, inhibits the proliferative response to exogenous T3. A mechanistic study further revealed that mH_2_O_2_, which was increased after T3 treatment, mediated the T3 proliferative response. Bogush et al. (2020) showed that exogenous T3 increases the cardiomyocyte endowment of P8 hearts, but the proliferative response—as assessed by cyclin A2 expression, phosphohistone-3 (H3P) labeling and lineage tracing studies—is confined to cardiomyocytes of the LV apex.

How does T3/mH_2_O_2_ signaling promote cardiomyocyte proliferation? Using *in vitro* and *in vivo* approaches, Tan et al. (2019) showed that T3-induced H_2_O_2_ stimulates insulin-like growth factor-1 (IGF-1) production. In turn, IGF-1 signaling increases the expression of wild-type p53-induced phosphatase 1 (WIP1), a checkpoint adaptor protein, which inhibits DDR signaling and inactivates p53. IGF-1 signaling simultaneously promotes ERK1/2 phosphorylation and the expression of cyclins that promote cell cycle entry and mitosis. Thus, T3/mH_2_O_2_-stimulated IGF-1 production not only inactivates cell cycle checkpoints by inhibiting DDR signaling, but simultaneously induces mitotic entry in cardiomyocytes through ERK1/2 activation.

## Mechanism-Based Regeneration Strategies

### Thyroid Hormone Signaling

T3 signaling through its receptors, TRα and TRβ, is usually associated with cardiomyocyte maturation. These effects of T3 prominently include: an increase in the α/β MHC ratio (Haddad et al., 2008); cardiomyocyte lengthening (Pantos et al., 2007); and, as discussed above, an increase in mitochondrial biosynthesis (Turrens, 2003; Marín-García, 2010), which transforms cardiomyocyte metabolism from glycolysis to OXPHOS (Puente et al., 2014; Hirose et al., 2019). More recently, however, T3 has been shown to induce cardiomyocyte proliferation (Naqvi et al., 2014; Figure 1). As detailed below, this effect is cell and context driven.

In neonatal (P1–P5) murine cardiomyocytes, *in vivo*, exogenous T3 increases the amount of cell cycle-promoting cyclins (e.g., D1, A2 and B1) and cardiomyocyte replication. The mechanism involves transcriptional induction of IGF-1 and IGF-1 receptor (IGF-1R) by TRα that leads to a robust increase in ERK phosphorylation (Tan et al., 2019).

In mice older than 2-weeks-of-age, exogenous T3 does not stimulate *in vivo* cardiomyocyte proliferation. This negative effect was surprising because T3 increases IGF-1 and IGF-R accumulation in adult cardiomyocytes as it does in neonatal cardiomyocytes. An important clue to explain this apparent paradox came from the finding that despite increased IGF-1/IGF-1R signaling by exogenous T3, increases in phospho-ERK were markedly attenuated. An in-depth analysis of this apparent discrepancy led to the discovery that after the neonatal period, cardiomyocytes begin to express dual specificity phosphatase-5 (DUSP5). DUSP5 is a phosphatase that differentially dephosphorylates phospho-ERK1/2 in the nucleus. When coupled with T3 administration, *in vivo* DUSP5 depletion, using siRNA, increases cardiomyocyte proliferation in adult mice (Bogush et al., 2020).

In the second week of life, DUSP5 levels in the LV increase in a distinct spatiotemporal fashion. In mice younger than P6, LV cardiomyocytes do not express DUSP5. After this period, DUSP5 expression increases gradually from the LV base to its apex over the next 7 days. Exogenous T3 stimulates cardiomyocyte cell cycle activation and replication in LV regions that are devoid of DUSP5 (Bogush et al., 2020).

After birth, developmental increases in circulating T3, in mice, occur at 2 distinct periods. First, there is a relatively small increase in circulating T3 that occurs at P4–P5 (Hirose et al., 2019). Then, at about P10–P12, there is a major increase in T3 biosynthesis that causes circulating T3 to increase to levels found in adults (Naqvi et al., 2014). Hirose et al. (2019) propose that a developmental increase in circulating T3 at ∼P4 causes neonatal cardiomyocytes to lose their proliferative capacity. In contrast, Naqvi et al. (2014) propose that the increase in circulating T3 at ∼P12 triggers the second wave of cardiomyocyte proliferation. The role of endogenous T3 in regulation of postnatal cardiomyocyte proliferation, therefore, remains controversial.

Naqvi et al. (2014) reported that cardiomyocyte numbers increase (by about 40%) during the first as well as the second week of life. Blocking endogenous T3 biosynthesis using short-term propylthiouracil (PTU) treatment (between P7 and P18) inhibited the second developmental increase in cardiomyocyte numbers. Hirose et al. (2019) reached an opposite conclusion. Long-term PTU treatment, starting from E13.5 to P14, caused an increase in ventricular cardiomyocyte numbers by P14. Maternal thyroid hormones have an important role in fetal heart development in rodents where placental transfer of maternal thyroid hormones has been shown to occur (Forhead and Fowden, 2014). Hirose et al. (2019) also used a genetic model. Here they inactivated TRα through expression of a dominant negative TRα mutant (*Myh6*-Cre;*Thraa*^*DN/+*^) in cardiomyocytes and found that it increased cardiomyocyte numbers in P14 hearts. The use of the *Myh6* promoter for these studies would suggest that such inactivation of TRα also occurs during the fetal period. The consequence on postnatal cardiomyocyte proliferative capacity of blocking T3 biosynthesis or TRα during the fetal period, is uncertain, but its understanding should be informative.

The effect of non-conditional genetic inactivation of cardiomyocyte TRα (*Myh6*-Cre;*Thra*^*DN/+*^) and exogenous T3 administration on cardiac regeneration has also been studied, but in distinct models of cardiac injury. Hirose et al. (2019) studied the role of TRα inactivation in ischemia-reperfusion (IR)-injured mice. At baseline, *Myh6*-Cre;*Thra*^*DN/+*^ mice had ∼40% larger hearts with 2-fold more cardiomyocytes compared to control mice (*Thra*^*DN/+*^ mice) and their LV posterior walls were thicker. IR injury resulted in similar-sized infarcts. At 4-week post-IR, *Myh6*-Cre;*Thra*^*DN/+*^ mice had reduced fibrosis and their LVEFs were ∼40-units higher than those of IR-injured controls (*Thra*^*DN/+*^). It is uncertain if the post-IR injury differences observed between *Myh6*-Cre;*Thra*^*DN/+*^ and *Thra*^*DN/+*^ mice are due to cardiomyocyte TRα inactivation or simply baseline differences in the LV geometry between these mice. Indeed, posterior wall thickness favorably affects myocardial energetics (Iismaa et al., 2018) and thus may have contributed to post-IR recovery between *Myh6*-Cre;*Thra*^*DN/+*^ and *Thra*^*DN/+*^ mice.

As discussed above, Bogush et al. (2020) showed that DUSP5 suppression, using siRNA, coupled with T3 treatment results in increased cardiomyocyte proliferation. Tan et al. (2021) studied DUSP5 siRNA + T3-induced cardiac regeneration in mice using the doxorubicin (cancer chemotherapeutic) injury model that is characterized by cardiomyocyte loss (Shi et al., 2010). Repeated doxorubicin administration results in cardiac injury leading to a ∼40 unit decrease in LVEF and an ∼45% increase in LV dilatation (LV volume at end-diastole). In a 3-week follow-up study, Tan et al. (2021) found that parenteral DUSP5 siRNA + T3 therapy rebuilt LV muscle by increasing cardiomyocyte numbers, which reversed LV dysfunction and prevented progressive chamber dilatation. Thus controlled ERK1/2 activation through transient DUSP5 siRNA + T3 therapy (Figure 1) induces preexisting cardiomyocyte proliferation to effect cardiac regeneration.

### MicroRNAs

MicroRNAs belong to a class of small (21–23 base pair size) non-coding RNAs that regulate expression of many genes, which can cause phenotype changes in cells, including cardiomyocytes. For example, if a microRNA inhibits cardiomyocyte proliferation through suppression of cell cycle activity, its blockade using an anti-miR might induce cardiomyocyte proliferation. Conversely, if a microRNA is known to induce cell cycle entry, administration of a mimic of this microRNA might induce cardiomyocyte proliferation (Figure 1).

MicroRNA-34a (miR-34a) levels are low during the first week of life in mouse hearts (Yang et al., 2015); a time when cardiomyocytes can still proliferate (Porrello et al., 2011; Naqvi et al., 2014). However, miR-34a levels increase after the neonatal period when cardiomyocyte replication is lost and remain high in adulthood. This suggested that miR-34a suppresses cardiomyocyte proliferation after the neonatal period and its blockade after MI injury might enhance regenerative repair through cardiomyocyte proliferation. To test this hypothesis, miR-34a was inhibited by administration of an anti-miR 6-h post-MI injury. Inhibition of miR-34a reduced cardiomyocyte apoptosis as measured by TUNEL staining and increased mitosis as measured by H3P labeling. Inhibition of miR-34a also reduced fibrotic scar area 3 days post-MI injury and improved cardiac function 7 days post-MI (Yang et al., 2015). In this study, cardiomyocyte replication was not established using unambiguous techniques such as genetic linage tracing or measurement of cardiomyocyte number. Furthermore, a substantial reduction in cardiomyocyte apoptosis by anti-miR therapy suggests that functional benefits of inhibiting miR-34a might be due to cardioprotection rather than cardiac regeneration.

In another study, Gao et al. (2019) injected miR-19a/19b mimics directly adjacent to the ligation site after coronary artery ligation and determined its impact on cardiac function over a 12-month follow-up period. Cardiac function measured at 2-weeks after injury was improved in miR-19a/19b treated hearts, which persisted for 12-months. The authors showed increased indices of cardiomyocyte mitosis in miR19a/19b treated hearts. However, the findings of a substantial reduction in cardiomyocyte and non-myocyte apoptosis as well as a reduction in inflammatory cells suggests that cardioprotection, rather than cardiomyocyte replication, accounts for the observed improvements in function.

Gabisonia et al. (2019) induced myocardial injury in pigs by subjecting them to 90 min of ischemia followed by reperfusion. This resulted in an ∼10 unit reduction in EF—that is, a mild injury. Intramyocardial injection of AAV6 viral vector carrying miR-199a at the time of reperfusion prevented further deterioration of EF and reduced infarct size compared to empty vector-injected control animals at 28-days post-IR injury. Cardiomyocyte proliferation was estimated using BrdU, Ki67, H3P and aurora kinase B labeling of cardiomyocytes. These markers showed substantially increased cell cycle activity in miR-199a-expressing hearts. However, miR-199a treatment resulted in increased mortality long-term due to uncontrolled cardiomyocyte proliferation (Gabisonia et al., 2019).

### Overexpression of Cell-Cycle Promoting Cyclins

Cell cycle-promoting cyclins such as D1, D2, A2 and B1 are expressed at high levels in cardiomyocytes during fetal life but decrease after birth. The adult mouse heart has undetectable levels of these cyclins and is also considered mitotically quiescent (Ikenishi et al., 2012). Based on these observations, some investigators hypothesized that a decrease in the levels of these cyclins in cardiomyocytes causes postnatal cell cycle arrest. They have, therefore, overexpressed cyclins to force proliferation of adult cardiomyocytes (Figure 1). Transgenic overexpression of cyclins D1, D2, D3 and A2 in cardiomyocytes from birth increased cardiomyocyte cell cycle entry (Soonpaa et al., 1997; Chaudhry et al., 2004; Pasumarthi et al., 2005). In addition, cyclin A2 overexpression by intramyocardial injection of an adenoviral vector at the time of MI improved cardiac output by ∼15% at 6-weeks follow-up (Woo et al., 2006). However, neither LVEF nor infarct size was determined immediately after MI to confirm that the treated and control groups had sustained the same degree of injury. While cell cycle activity was determined (by H3P labeling), the report provided no definitive evidence for meaningful cardiomyocyte replication (such as genetic lineage tracing or measuring cardiomyocyte numbers). Cyclin D2-overexpressing mice also had smaller infarcts after MI injury (Pasumarthi et al., 2005). However, ventricular muscle thickness is increased in these mice compared to wildtype littermates, even before MI surgery, due to continued cardiomyocyte proliferation from birth. Increased muscle thickness favorably affects myocardial energetics (Iismaa et al., 2018) and could have accounted for the reduced infarct size observed in cyclin D2 overexpressing mice. These studies also revealed that cyclin overexpression leads to enlarged hearts that are initially hypercontractile but then progress to become hypocontractile (Chaudhry et al., 2004), which could mean that this regenerative strategy might not be safe long-term.

### Extracellular Matrix Proteins

Non-myocytes in the cardiac interstitium secrete proteins that form a dynamic three-dimensional network called the ECM, which provides a structural scaffold that offers mechanical support to preserve cardiac geometry. The ECM also functions as a reservoir for growth factors and proteins secreted by cardiomyocytes and non-myocytes. ECM also plays a critical role in cardiac homeostasis by facilitating intercellular communication and signal transduction to modulate cellular responses, both physiologically and in response to injury. Although growth factors and proteins released by cardiomyocytes, endothelial cells and inflammatory cells become part of the matrix, the cardiac ECM is primarily formed and modified during health and after a myocardial injury by the proteins released by cardiac fibroblasts (Fan et al., 2012). These proteins include, but are not limited to, collagens, growth factors, proteases, periostin, agrin, fibronectin, osteopontin, thrombospondins, and laminins. Thus, ECM composition is continually regulated by synthesis and degradation by proteases in the cardiac interstitium. These proteins also impact the structural properties of the matrix. For example, stiffness of the matrix may alter cell movement, survival and proliferation (Wells, 2008). Manipulation of ECM proteins can impact cell phenotype by altering signal transduction by integrins; receptors for these ECM proteins. For example, the RGD motif and amino acids surrounding this motif that are present in most of ECM proteins determine binding of the ECM proteins to integrins, which activates intracellular signal transduction (Ruoslahti, 1996; Chen et al., 1997). ECM proteins are also implicated in cell cycle pathways impacting cardiac regeneration from lower vertebrates to mammals.

In a zebrafish cardiac regeneration model, Poss and co-investigators used a proteomics approach to show that fibronectin (an ECM protein) is associated with zebrafish heart regeneration and further, that regeneration was abolished with fibronectin loss-of-function mutations (Wang et al., 2013).

Periostin is an ECM protein that promotes cell survival through AKT signaling (Bao et al., 2004). Importantly, in mice, its expression increases after acute MI injury where it promotes cardiac healing through its interaction with fibroblasts to modulate collagen fibril formation to prevent cardiac rupture (Shimazaki et al., 2008). Kühn et al. (2007) showed that treatment of cardiomyocytes with periostin induces proliferation of adult cardiomyocytes *in vitro* and *in vivo* in healthy mouse hearts. Furthermore, while MI injury reduced LVEF by ∼12%, periostin applied as a cardiac patch at the time of MI injury increased LVEF by ∼13-units). Post-MI cardiomyocyte cell cycle activity was measured using BrdU and H3P immunohistochemistry while cardiomyocyte replication was indirectly estimated from counting cardiomyocyte nuclei.

Tzahor’s group used proteomics to identify agrin as an ECM component present in regenerating mouse neonatal ECM but absent from post-neonatal non-regenerative ECM. They showed that agrin promotes cardiomyocyte proliferation through YAP-ERK signaling (Figure 1). Importantly, intramyocardial injection of agrin at the time of MI or IR injury improved LVEF over 4-week follow-up in mouse and pig infarction models (Bassat et al., 2017; Baehr et al., 2020). However, in pigs, an important functional benefit of agrin therapy was reduced apoptosis (Baehr et al., 2020).

### Neuregulin

NRG1 is a member of the epidermal growth factor family that activates membrane-bound tyrosine kinase receptors, ErbB4 or ErbB3, when they heterodimerize with ErbB2. A recombinant NRG1 peptide improves cardiac function and survival in ischemic, dilated and viral cardiomyopathies (Liu et al., 2006). However, the mechanism for these beneficial effects remained unclear. It was of interest, therefore, when Bersell et al. (2009) showed that administration of recombinant NRG1 stimulates neonatal cardiomyocyte proliferation *in vitro.* In healthy young adult mice, overexpression of ErbB4 induced S-phase entry in mononuclear cardiomyocytes. They also showed that recombinant NRG1 administration increased cardiomyocyte cell cycle entry as well as the number of cardiomyocytes. Using genetic fate mapping, the source of new cardiomyocytes was found not to be stem cells but replication of preexisting cardiomyocytes. Importantly, daily injections of recombinant NRG1 for 12-weeks, starting 1-week post-MI, reduced scar size, improved cardiac function, and blunted cardiomyocyte hypertrophy. However, subsequently Field and co-investigators reported that recombinant NRG1 therapy does not stimulate cardiomyocyte proliferation in healthy or MI-injured mouse hearts (Reuter et al., 2014). Nevertheless, Poss and co-investigators showed that NRG1 is induced in the injured zebrafish heart and that blocking ErbB2 impairs regenerative repair of the heart. Furthermore, Tzahor and co-investigators found that ErbB2 expression falls rapidly after birth and is undetectable by the end of first week of mouse life. Overexpression of a constitutively active form of the ErbB2 receptor (caERBB2) in young adult mice resulted in robust cardiomyocyte proliferation. Importantly, ERK1/2 activation was necessary for this robust proliferative response. Overt proliferation was associated with the development of abnormally large hearts, which led to increased mortality (D’Uva et al., 2015). Thus, activation of NRG1/ERBB2/ERK1/2 signaling pathway potently induces cardiomyocyte proliferation (Figure 1) and it needs to be controlled to avoid cardiomegaly.

Polizzotti et al. (2015) administered recombinant NRG1 to mouse pups at birth (1 day before cryoinjury, performed on P1 and continued daily for the next 34 days. This protocol resulted in a sustained improvement in heart function up to one-month after cessation of therapy. However, consistent with the downregulation of the receptor for NRG1 (ErbB2) immediately after birth, this benefit was lost if NRG1 therapy was started at P5 (D’Uva et al., 2015). This suggests a narrow therapeutic window for recombinant NRG1 therapy. Tzahor and colleagues overcame this problem by overexpression of a mutated form of the ErbB2 receptor (CaERBB2). CaERBB2 is constitutively active and thus does not require its ligand, NRG1 and also induces cardiomyocyte proliferation also through ERK1/2 activation (D’Uva et al., 2015; Aharonov et al., 2020; Figure 1). Overexpressing caERBB2 in mice from 3-6-weeks post-MI initially transiently reduced stroke volume and cardiac output for 3-weeks. This depression in cardiac function was ascribed to cardiomyocyte dedifferentiation and hypertrophy. Cardiac contractile function then improved over the next 3-weeks associated with redifferentiation and reversal of cardiomyocyte hypertrophy. Scar size was also reduced (Aharonov et al., 2020) demonstrating the regenerative potential of NRG1/ErbB2 signaling pathway.

### p38 and Meis1

Mechanistic studies of heart development have identified additional molecular pathways/targets regulating the postnatal cardiomyocyte cell cycle activity. Mitogen-activated protein (MAP) kinases comprise a large family of proteins and act as a hub for diverse signal transduction pathways and play a key role in several biological processes. Fibroblast growth factor (FGF) family members are also involved in numerous biological processes possess such as being a mitogen and antiapoptotic. Pharmacological inhibition of p38 MAP kinase using a p38 inhibitor (SB203580HCl) together with FGF1 administration induces proliferation of large adult binuclear cardiomyocytes *in vitro* (Engel et al., 2005). Importantly, intramyocardial injection of FGF1 combined with intraperitoneal injection of SB203580HCl at the time of MI injury prevented LV contractile dysfunction 24 h after MI injury. This therapy also reduced scar size and prevented LV dilatation when treatment was continued for 1-month post-MI injury (Engel et al., 2006). FGF1 stimulation or p38 inhibition has been shown to be cardioprotective post-MI injury by promoting cardiomyocyte survival (Palmen et al., 2004; Kaiser et al., 2004). Given that treatment of mice with FGF1 or SB203580HCl monotherapy similarly attenuated LV dysfunction to that observed in animals treated with FGF1 plus the p38 inhibitor, these benefits may be attributed to antiapoptotic effects of FGF1 signaling and/or p38 inhibition as suggested by Engel et al. (2006).

Meis1 is a member of the TALE (three amino acid loop extension) family of homeodomain transcription factors with an essential role in heart development (Paige et al., 2012; Wamstad et al., 2012). In the mouse heart, Meis1 expression increases after birth around P7, the time of cardiomyocyte cell cycle arrest. Thereafter, Meis1 expression remains high and has been suggested to maintain adult cardiomyocytes in a state of cell cycle arrest (Mahmoud et al., 2013). Genetic knockdown of Meis1 during adulthood increases cardiomyocyte cell cycle entry (Mahmoud et al., 2013). Based on these findings, targeting Meis1 was proposed as a therapeutic approach for regenerating injured adult hearts (Mahmoud et al., 2013) but, as yet, has not been tested.

### Hypoxia

Placing the MI-injured hearts in a low oxygen environment was recently proposed as a therapeutic strategy to stimulate cardiomyocyte regeneration for myocardial repair post-MI (Nakada et al., 2017). Mice subjected to either hypoxia or normoxia for 2 weeks after MI injury, were gradually returned over 1 week to normoxia, and then cardiac function determined. While EF in the normoxia group did not improve, mice treated with hypoxia had an ∼15 unit increase in EF. However, because hypoxia was started within 1 week of MI-injury (before scar maturation and remodeling of heart), the impact of hypoxia on adverse LV remodeling, such as infarct expansion and LV dilatation is not known. Also, because the study was terminated immediately after the hypoxia treatment, it is unknown if 2 weeks of hypoxia therapy has a long-term impact on reverse LV remodeling. This study is based on the concept that immediately after birth transition from the fetal hypoxic to a normoxic environment causes cardiomyocyte cell cycle arrest due to reactive oxygen species (ROS) generated by mitochondrial OXPHOS (Puente et al., 2014). Therefore, use of NAC (a non-specific antioxidant), increases cardiomyocyte proliferation by inhibiting ROS-induced DNA damage (Puente et al., 2014). However, it is not clear how hypoxia therapy will be translated to ischemic heart patients. One way of translating this concept would be to give antioxidants to patients with MI injury, but studies using antioxidants in the setting of MI injury have not shown any benefit (Yellon and Hausenloy, 2007).

### Hippo-YAP Pathway

Hippo kinases were first discovered in the fruit fly, *Drosophila melanogaster*, where an inactivating mutation in the gene encoding Hippo, the Drosophila Ste20 family kinase (dMST), showed that Hippo functions as a tumor suppressor by restricting cell proliferation and promoting apoptosis (Jia et al., 2003). The Hippo kinase pathway is evolutionary conserved in mammals, with Hippo orthologs, the Ste20-like protein kinases (Mst1/2) interacting with the Salvador homolog 1, Salv1, to phosphorylate and activate large tumor suppressor homologs (Lats1/2). Lats1/2 in turn phosphorylates the transcriptional co-activator, yes-associated protein 1 (YAP1). YAP1 works in collaboration with the transcription coactivator PDZ-binding motif (TAZ) to mediate gene transcription programs including but not limited to cell proliferation. This phosphorylation of YAP by Lats1/2 inactivates Hippo/YAP signaling by preventing translocation of the YAP/TAZ complex into the nucleus. Phosphorylation of YAP by Hippo kinases during early heart development restricted its transcriptional activity to preclude heart overgrowth (Heallen et al., 2011). Also, many studies have shown that YAP1 has an obligatory role in cardiomyocyte replication, involving distinct proliferative pathways, such as Wnt/β-catenin, IGF-1R/β-catenin, Pi3kcb or epigenetic reprogramming (Xin et al., 2011; von Gise et al., 2012; Lin et al., 2015; Monroe et al., 2019).

Cardiomyocytes become mitotically quiescent during the early postnatal period. However, reactivation of YAP signaling through genetic approaches induces cardiomyocyte proliferation in adults. Mutating YAP at serine 127 to alanine produces a gain-of-function phenotype. Overexpression of YAP^S127A^ protein by intramyocardial injection of a viral vector encoding YAP^S127A^, at the time of MI injury, improved cardiac function and survival. Cardiomyocyte proliferation was confirmed by genetic lineage tracing (Lin et al., 2014).

There are five serine residues in YAP that can potentially be phosphorylated by Hippo kinases to inhibit YAP transcriptional activity (Monroe et al., 2019). Mutating all five serine residues to alanine generates a highly active form of YAP, designated as YAP^5SA^. Conditional overexpression of YAP^5SA^ in adult cardiomyocytes produced overt proliferation of these cells resulting in LV chamber occlusion and lethality within 4 days (Monroe et al., 2019).

Hippo pathway component, Salv1, cooperates with Hippo kinases to inactivate YAP. Conditional inactivation of Salv1 in adult cardiomyocytes using αMHC-mcm;Salv^f/f^ (SalvCKO) mouse prevents YAP inactivation through its phosphorylation by Hippo kinases (Leach et al., 2017). Preclinical studies with SalvCKO mice then tested the hypothesis if MI injury-induced LV dysfunction could be reversed by inactivation of the negative regulator of YAP. LVEF before MI injury was about 64%. After MI injury, EF decreased to about 36%. Three weeks post-MI, mice were either treated with tamoxifen to conditionally delete the Salv1 gene from cardiomyocytes. This deletion reversed MI-induced LV dysfunction. Genetic lineage tracing confirmed cardiac regeneration through replication of preexisting cardiomyocytes. Recently, the efficacy of viral vector-mediated *Salv1* knockdown has been tested in pig hearts subjected to MI (Liu et al., 2021). After MI injury, LVEF was reduced from ∼60% to low 40%. Following intramyocardial injection of AAV9-Sav-shRNA 2-weeks after MI-injury, LVEF improved only marginally (by ∼10 units).

## Assessment of Extant Preclinical Cardiac Regeneration Research

Extant studies have examined regenerative therapies in small animal (mainly rodent) models as discussed above (Table 1). However, rodent model has its strengths and limitations. Preclinical testing in a small animal model has the advantage of being cost effective and rapid. Importantly, it is easier to manage larger cohorts of animals to have ample statistical power for the study and thus the conclusions drawn from the study will be well supported. However, rodents have differences in cardiac physiology than humans such as higher heart rate. Furthermore, preclinical studies in small animals show larger functional benefits than in large animals which could be related to differences in the cardiac physiology between rodents and large animals. In this respect larger animals (especially the pig) are considered to have higher physiological relevance to humans (Lindsey et al., 2018) and have consistently shown similar drug responses as in human clinical trials (Zwetsloot et al., 2016). However, studies in pigs are expensive. Moreover, most studies in large animals are carried out in an acute MI model with mild to moderate MI injury. Large animal models with chronic severe LV dysfunction are not well established. These challenges need to be addressed for effective translation of these therapies in humans.

**TABLE 1:** Summary of mechanism-based regenerative strategies in preclinical studies.

**Target pathway**	**Species**	**Age at MI**	**Heart failure severity**	**Time therapy started**	**Follow-up duration**	**CM proliferation estimation**	**References**
Cell cycle (Cyclin D2)	Mouse	Young adult	Not known	Embryonic life	5-months	CM density in histological sections	Pasumarthi et al., 2005
Cell cycle (Cyclin A2)	Rat	Young adult	Non-known	At the time of MI	6-weeks	BrdU, PCNA and H3P labeling	Woo et al., 2006
ECM (Periostin)	Mouse	Young adult	Mild	At the time of MI	12-weeks	BrdU, H3P labeling, CM nuclei count	Kühn et al., 2007
ECM (Agrin)	Mouse	Young adult	Mild to moderate	At the time of MI	4-weeks	BrdU, Ki67 and Aurkb B labeling	Bassat et al., 2017
ECM (Agrin)	Pig	Young adult	Moderate	At the time of MI	4-weeks	BrdU labeling	Baehr et al., 2020
miR-34a	Mouse	Young adult	Moderate	6-h post-MI	1-week	H3P labeling	Yang et al., 2015
miR-19a/19b	Mouse	Young adult	Not known	At the time of MI	12-months	Direct CM counting, EdU, H3P and Aurkb labeling	Gao et al., 2019
miR-199a	Pig	Young adult	Mild	At the time of MI	4-weeks	BrdU, H3P labeling, CM number by CM nuclei	Gabisonia et al., 2019
TRα	Mouse	Young adult	Mild	Embryonic life	4-weeks	EdU, H3P and Aurkb labeling	Hirose et al., 2019
Neuregulin 1	Mouse	Young adult	Moderate	1-week post-MI	12-weeks	BrdU, H3P, Aurkb labeling, CM number by CM nuclei	Bersell et al., 2009
ERBB2	Mouse	Young adult	Mild	At the time of MI	2-weeks	Ki67 and Aurkb B labeling	D’Uva et al., 2015
ERBB2	Mouse	Young adult	Moderate	3-weeks post-MI	3-weeks	RNAseq–Cyclin D1, ERK, YAP	Aharonov et al., 2020
p38/FGF1	Rat	Young adult	Moderate	At the time of MI	8-weeks	Cyclin A2 and H3P labeling	Engel et al., 2006
Hypoxia/DDR	Mouse	Young adult	Moderate	1-week post-MI	2-weeks	CM density, BrdU, H3P and Aurkb labeling	Nakada et al., 2017
Hippo/Yap (Salv1)	Mouse	Young adult	Mild to Moderate	3-week post-MI	6-weeks	EdU and H3P labeling, Lineage tracing	Leach et al., 2017
Hippo/Yap (YAP^S127A^)	Mouse	Young adult	Not known	1-week post-MI	4-weeks	EdU and H3P labeling, Lineage tracing	Lin et al., 2014
Hippo/Yap (Salv1)	Pig	Juvenile	Moderate	2-week post-MI	12-weeks	CM density, EdU, H3P and Aurkb labeling	Liu et al., 2021
DUSP5 and T3/IGF-1	Mouse	Young adult	Severe	6-weeks after first doxorubicin exposure	3-weeks	Lineage tracing, CM counting and CM density	Tan et al., 2021

*Heart failure severity is binned in 3 groups–Mild (injury induces a reduction in LVEF of 10–20 percentage points), Moderate (LVEF is reduced between 20–35 percentage points) and Severe (LVEF reduction > 35 percentage points with LV dilatation). According to the guidelines by the American College of Cardiology, severe LV dysfunction for patients in heart failure is defined as a decrease in LVEF of > 30-units. This is based on a normal LVEF of 60% in adult humans (American College of Cardiology: https://www.acc.org/tools-and-practice-support/clinical-toolkits/heart-failure-practice-solutions/left-ventricular-ejection-fraction-lvef-assessment-outpatient-setting). MI: myocardial infarction injury, LVEF: left ventricular ejection fraction, CM: cardiomyocyte, BrdU: 5-bromodeoxyuridine, EdU: 5-ethynyl-2′-deoxyuridine, H3P: phosphohistone H3, Aurkb: aurora kinase B, PCNA: proliferating cell nuclear antigen.*

Importantly, it remains unclear if the functional benefits that are observed with several regenerative therapy protocols targeting the various molecular pathways, discussed above, are due to *bona fide* cardiac regeneration resulting from induced proliferation of preexisting cardiomyocytes or to other mechanisms, such as decreased cardiomyocyte apoptosis or improved cardiac energetics. Here we provide a perspective on extant preclinical research using mechanism-based regenerative strategies (Table 1).

### Estimation of Post-therapy Cardiomyocyte Proliferation

MI-injury results in substantial loss of functional cardiomyocytes triggering heart failure. Therefore, proliferation of spared cardiomyocytes is the key objective of mechanism-based strategies to effect regenerative repair of infarcted hearts. Assessment of cell cycle markers such as BrdU and EdU-labeling provides evidence of S-phase entry of cardiomyocytes. Aurora kinase B and H3P are widely used markers of mitosis. However, these markers do not provide evidence for cardiomyocyte replication because after cardiomyocytes enter the cell cycle, they can follow distinct trajectories. For example, the cell cycle can terminate prematurely leading to increased ploidy or multinucleation. Labeling efficiency of these markers is uncertain and immunohistochemical assessments of cell cycle markers merely provide a snapshot of cell cycle activity at the time of tissue procurement.

Genetic lineage tracing, by contrast, shows unambiguous replication of preexisting cardiomyocytes (Porrello et al., 2011; Lin et al., 2014; Leach et al., 2017; Tan et al., 2021). However, this technique does not quantify the extent of cardiomyocyte number expansion. Design-based stereology provides an estimate of cardiomyocyte number. The term design-based describes a technique in stereology in which sampling is independent of the size, shape, spatial orientation, and spatial distribution of the geometrical features to be investigated. Random sampling assumes that cardiomyocyte proliferation occurs homogeneously in the LV and that there are no location-based differences in cardiomyocyte morphology. In adult hearts, cardiomyocytes adopt a variety of shapes: simple elongated cylinders or branched. Importantly, cardiomyocytes in different anatomical locations of the heart may not respond uniformly to therapeutic stimuli. For example, cardiomyocytes in the infarct border-zone might respond differently to those in remote zone. Moreover, post-neonatal cardiomyocytes from the LV apex were recently shown to be differentially sensitive to a mitogenic stimulus (Bogush et al., 2020). A further consideration with respect to stereology is that tissue shrinkage, if not studied directly, has the potential to variably impact total cardiomyocyte number calculation between hearts and between different LV regions. Such differences in random tissue sampling might explain why, using the same collection of human hearts, two laboratories reported divergent findings. Mollova et al. (2013) showed a 3.4-fold increase in cardiomyocyte number in humans between birth and late adolescence, while Bergmann et al. (2015) reported no change in cardiomyocyte numbers. This difference occurred even though both laboratories provide evidence for robust cardiomyocyte proliferation during this developmental period.

Another technique to indirectly estimate the number of cardiomyocytes involves measuring the total number of cardiomyocyte nuclei after a regenerative therapy (Kühn et al., 2007; Bersell et al., 2009). However, induced multinucleation in cardiomyocytes would make this technique problematic. For example, cyclin D1 overexpression in cardiomyocytes causes profound multinucleation (Soonpaa et al., 1997). Direct measurement of absolute cardiomyocyte number by disaggregating the ventricular myocardium is the most widely used technique to show definitive cardiomyocyte proliferation (Mahmoud et al., 2013; Naqvi et al., 2014; Puente et al., 2014; Nakada et al., 2017; Tan et al., 2021). This technique requires optimization to achieve a high level of digestion efficiency > 97% (e.g., Bogush et al., 2020). Because enzymatic heart disaggregation requires a patent coronary artery for perfusion with collagenase, it cannot easily be used to determine cardiomyocytes numbers in post-MI hearts where a major branch of the coronary tree has been permanently ligated. However, in other forms of injury that do not involve coronary artery occlusion (e.g., anthracycline induced heart failure) post-therapy changes in cardiomyocyte number have been reported (Tan et al., 2021).

It is important, therefore, that preclinical studies evaluating bona fide cardiomyocyte replication be conducted using techniques, such as genetic lineage tracing and direct cardiomyocyte number quantification, wherever possible, as reported by some investigators (e.g., Lin et al., 2014; Leach et al., 2017; Tan et al., 2021) (Table 1).

### Cardioprotection Versus Cardiac Regeneration

Both, ischemia (Sutton and Sharpe, 2000) and reperfusion injury (Wu et al., 2018) trigger cardiomyocyte death. Therefore, cardioprotective therapies are usually given immediately after MI or IR injury to reduce cardiomyocyte death with the aim of limiting infarct size, LV dysfunction and adverse LV remodeling (e.g., Piot et al., 2008; Dong et al., 2009; Mewton et al., 2010). Regenerative pathways often intersect with cardioprotective pathways. YAP signaling simultaneously promotes cardiomyocyte proliferation and survival *in vivo* (Lin et al., 2015). To ensure that a therapy is not merely cardioprotective, regenerative therapies must be given after the initial phase of cardiomyocyte apoptosis has ceased, which may not occur until about 3 or 4 days after the ischemic injury (Tejada et al., 2016). However, many cardiac regenerative therapies tested have been given either at the time of, or immediately after MI (Table 1). For example, Gao et al. (2019) showed that miR-19a/19b therapy given at the time of MI increased cardiomyocyte mitosis along with a substantial reduction in apoptosis. In this situation, cardioprotection might be an important component of the improvement in LV contractile function. Similarly, the finding that agrin therapy given at the time of IR injury reduced apoptosis (Baehr et al., 2020), reiterates the need to study the contribution of cardioprotective mechanisms toward the overall improvement in LV contractile function, if the therapy is given before or within the first week after MI injury.

### Preinjury Differences in Cardiac Phenotypes

Some preclinical studies, involving genetic models, show that, at baseline, the heart has more cardiomyocytes and a thicker LV posterior wall (e.g., Pasumarthi et al., 2005; Hirose et al., 2019). It is not surprising that these mice fair better after MI injury because these mice have increased muscle thickness, which favorably affects myocardial energetics (Iismaa et al., 2018). Such differences between phenotypes must be considered before drawing conclusions about the mechanism of regenerative therapies in genetically modified mice.

## Conclusion

### Long-Term Safety and Efficacy

Most preclinical reports based on induction of cardiomyocyte proliferation have not conducted long-term follow-up studies after regenerative therapy (Table 2). This is especially important because regenerative therapies have the potential to change the phenotype of the cardiomyocytes. For example, stimulation of cardiomyocytes to proliferate cause them to dedifferentiate resulting in sarcomeric disassembly (Bersell et al., 2009) and reversion to a fetal-like state characterized by repression of the fast-contractile myosin isoform, αMHC (Monroe et al., 2019). Dedifferentiation is also linked to adverse cardiac remodeling (Kubin et al., 2011). Furthermore, dedifferentiation blunts ATP production by switching from energy-efficient OXPHOS to glycolysis. This could be detrimental for an organ like the heart which has a high energy demand. Indeed, energy starvation is a feature of heart failure (Ventura-Clapier et al., 2004) and a decrease in the α/β - MHC ratio depresses LV contractility and increases disease severity (Krenz and Robbins, 2004). Furthermore, dedifferentiated cardiomyocytes might be a substrate for arrhythmias if these cardiomyocytes do not electrically couple to existing cardiomyocytes. Thus, in preclinical follow-up studies, it is important to show that long-term regenerative repair does not cause electrical conduction dysfunction and arrhythmias. A recent report underscored these concerns by showing that substantial decreases in LV contractile function occurred after genetic overexpression of caERBB2 post-MI injury (Aharonov et al., 2020). This decrease in LV function was ascribed to cardiomyocyte dedifferentiation and hypertrophy, which could be a reason why this therapy is ineffective in repairing severely injured hearts (Aharonov et al., 2020). In severe heart failure patients, further deterioration of LV contractile function, even if it is transient, may be fatal. Thus, there is a need for strategies that either do not or only minimally repress OXPHOS or induce cardiomyocyte dedifferentiation and hypertrophy.

**TABLE 2:** Potential translation strategies based on preclinical research.

**Preclinical therapy**	**Preclinical safety (test time)**	**References**	**Potential therapeutic drug and delivery route in humans**
Cyclin D2	Not tested	Pasumarthi et al., 2005	Cyclin D2 viral vector/intramyocardial injection
p38 inhibition	Not tested	Engel et al., 2006	p38 inhibitor/intramyocardial injection
Cyclin A2	Not tested	Woo et al., 2006	Cyclin A2 viral vector/intramyocardial injection
Periostin	Not tested	Kühn et al., 2007	Recombinant periostin/epicardial patch
Neuregulin 1	Not tested	Bersell et al., 2009	Recombinant neuregulin 1/subcutaneous
YAP^S127A^	Not tested	Lin et al., 2014	YAP^S127A^ viral vector/intramyocardial injection
miR-34a	Not tested	Yang et al., 2015	miR-34a/intravenous
CaERBB2		D’Uva et al., 2015	CaERBB2 viral vector/intramyocardial injection
Hypoxia	Not tested	Nakada et al., 2017	Antioxidant/oral
Salv1	Not tested	Leach et al., 2017	Salv1 viral vector/intramyocardial injection
Agrin	Not tested	Bassat et al., 2017	Recombinant agrin/intramyocardial injection
miR-19a/19b	Safe (12 months)	Gao et al., 2019	miR-19a/19b/intramyocardial injection
miR-199a	Unsafe	Gabisonia et al., 2019	—
TRα inhibition	Not tested	Hirose et al., 2019	T3 biosynthesis inhibitor/oral
Salv1	Not tested	Liu et al., 2021	Salv1 viral vector/intramyocardial injection
DUSP5 inhibition + T3	Not tested	Tan et al., 2021	T3 + DUSP5 inhibitor/oral-intravenous

Importantly, undesirable effects of regenerative therapy, such as uncontrolled cardiomyocyte proliferation, could adversely impact LV remodeling long term. For example, overexpression of cyclins can produce overt cardiomyocyte proliferation causing cardiomegaly; these hearts initially show hypercontractility but eventually deteriorate to become hypocontractile (Chaudhry et al., 2004). Recently, Monroe et al. (2019) overexpressed a mitogenic protein and showed that uncontrolled cardiomyocyte proliferation caused overgrowth of the LV free wall, leading to outflow tract obstruction with consequent reductions in cardiac output and increased mortality. Using a microRNA approach, Gabisonia et al. (2019) showed that regenerative therapy worked well in the short term; in the long term it increased mortality, which was ascribed to uncontrolled cardiomyocyte proliferation. Thus, it is important that regenerative therapies be evaluated over several months, rather than weeks, to ensure that they have long-term efficacy and safety.

### Translatability

Current regenerative approaches have limited or complicated paths towards effective translation. Some use a genetic approach to overexpress a protein from embryonic life onwards, others use viral vectors for delivery of genes into cardiomyocytes, while others still inject proteins (e.g., FGF1, agrin) directly into the LV wall (Table 2). In contrast to these approaches, development of small molecule(s) that when given parenterally can stably build LV muscle through controlled cardiomyocyte proliferation, would greatly enhance the translational potential of regenerative therapies.

### Efficacy of Regenerative Therapy in Reversing Chronic Severe Heart Failure

Post-MI wound healing can be divided into 3 stages: inflammation/necrosis, fibrosis/proliferation, and long-term remodeling/maturation (Richardson et al., 2015). The inflammatory phase occurs over the first few days. In the second phase, fibroblasts proliferate, and collagen accumulation begins by 7–14 days post-MI. Scar formation is complete at 21 days post-MI (Yang et al., 2002). In the final phase, which lasts several weeks, the scar matures via a steady increase in collagen crosslinking (Richardson et al., 2015). In this final phase, also, the LV continues to dilate, causing infarct expansion because of LV wall thinning. Thus, chronic (≥ 1-month) post-MI hearts are very different from acute (1-day-to-1-week) post-MI hearts in that the injury-initiated immune response, which is a critical component of the early regenerative process (Epelman et al., 2015), is no longer operative. Extant cardiac regenerative therapies have been given either at the time of, or soon after MI (Table 1). In a few cases, regenerative therapy has been given in a more chronically-injured heart (about 3 to 6 weeks after injury) (Leach et al., 2017; Aharonov et al., 2020; Tan et al., 2021).

LVEF and LV end-systolic volume have been shown to be the strongest predictors of cardiac death following a MI (White et al., 1987; Roes et al., 2007); LV infarct size negatively correlates with LVEF. This correlation is only observed after MI injury that increases infarct size by > 15% (Pride et al., 2010); a finding that highlights the importance of the injury severity for the development of post-MI LV dysfunction. Severe MI injury with larger loss of myocardial muscle results in severe adverse remodeling (e.g., LV dilatation), which determines heart failure severity (Gaudron et al., 1993). A review of the existing preclinical studies suggest that most regenerative therapies have been tested following mild to moderate MI injury as assessed by post-MI LVEF and LV dilatation (Table 1). For example, in the study by Leach et al. (2017), the MI injury was moderate; it only resulted in a 28-unti decrease in LVEF without LV dilatation. Similarly, Aharonov et al. (2020) showed that their cardiac regenerative therapy worked within a threshold of functional loss that did not lead to severe heart failure. Gabisonia et al. (2019) also used a large animal model where MI resulted in a decrease in EF of only ∼10 units. Thus, future preclinical studies need to adequately address the applicability of the therapy for treating patients with chronic severe heart failure.

### Ability of a Regenerative Therapy to Impact Quality-of-Life

From a patient’s perspective, shortness of breath and fatigue are worrisome symptoms of heart failure and a cause for panic in many such patients; these symptoms increase hospitalizations (McDermott et al., 2017). Consequently, patients with a severely remodeled heart avoid physical activity and experience significant deterioration in quality-of-life, as a result of depression, the feeling of hostility toward others and difficulty in performing daily activities (Dracup et al., 1992; Bennet et al., 2002; Hobbs et al., 2002; Cai et al., 2019). There is no therapy currently available to reverse the trajectory of such patients. Regenerative repair by improving contractile function needs to enhance quality-of-life. However, in extant preclinical animal studies that have tested regenerative therapies, quality-of-life assessments are rarely undertaken. In small animal studies, measures of quality-of-life include assessments of grooming status, movement, and ability to perform exercise, including endurance testing.

### Aging

Almost all preclinical studies are performed in young adult mice (between 5 to 12 weeks old) (Table 1) but, in humans, heart failure mostly afflicts older adults. Furthermore, aging is associated with an increase in the number of heart disease deaths (Sidney et al., 2018). Given that older adults in the US are projected to increase by > 40% in the next 10 years, heart failure therapies must be effective for all age groups. Thus, it is important to know if a regenerative therapy is as effective in triggering cardiomyogenesis in older mice as it does in young adults. Cardiomyogenesis requires cardiomyocyte proliferation concurrent with effective neovascularization (Marín-Juez et al., 2016; Ingason et al., 2018). However, aging impairs neovascularization (Lähteenvuo and Rosenzweig, 2012; Ungvari et al., 2018). Thus, it is important to understand if a regenerative therapy can induce effective cardiomyogenesis by not only inducing cardiomyocyte proliferation but also neovascularization in aged mice. Recently, microRNA-130a therapy was shown to rescue aging-related impaired neovascularization in ischemic muscle by suppressing MEOX2 and HOXA5 gene expression (Dhahri et al., 2020). Thus microRNA-130a co-administration with a regenerative therapy may enhance the regenerative repair in aged mice.

In summary, although initial proof-of-concept cardiac regenerative studies show promise, critical barriers, apart from stimulating cardiomyogenesis, must also be addressed. A therapy that regenerates mildly injured hearts (e.g., Leach et al., 2017; Aharonov et al., 2020) might not be effective in severely injured hearts with marked LV remodeling. It is possible that regenerative therapy approaches to treating severely injured hearts might need to combinatorically target pathways that remodel scar, as well as limit inflammation and enhance neovascularization as well as inducing cardiomyocyte proliferation.

## Author Contributions

NN and AH researched the literature and wrote the manuscript with input from RG and SI. All authors contributed to the article and approved the submitted version.

## Conflict of Interest

The authors declare that the research was conducted in the absence of any commercial or financial relationships that could be construed as a potential conflict of interest.

## Publisher’s Note

All claims expressed in this article are solely those of the authors and do not necessarily represent those of their affiliated organizations, or those of the publisher, the editors and the reviewers. Any product that may be evaluated in this article, or claim that may be made by its manufacturer, is not guaranteed or endorsed by the publisher.

## References

[B1] Aharonov A. Shakked A. Umansky K. B. Savidor A. Genzelinakh A. Kain D. (2020). ERBB2 drives YAP activation and EMT-like processes during cardiac regeneration. *Nat. Cell Biol.* 22 1346–1356. 10.1038/s41556-020-00588-4 33046882

[B2] Alkass K. Panula J. Westman M. Wu T. D. Guerquin-Kern J. L. Bergmann O. (2015). No evidence for cardiomyocyte number expansion in preadolescent mice. *Cell* 163 1026–1036. 10.1016/j.cell.2015.10.035 26544945

[B3] Baehr A. Umansky K. B. Bassat E. Jurisch V. Klett K. Bozoglu T. (2020). Agrin promotes coordinated therapeutic processes leading to improved cardiac repair in pigs. *Circulation* 142 868–881. 10.1161/CIRCULATIONAHA.119.045116 32508131

[B4] Bao S. Ouyang G. Bai X. Huang Z. Ma C. Liu M. (2004). Periostin potently promotes metastatic growth of colon cancer by augmenting cell survival via the Akt/PKB pathway. *Cancer Cell* 5 329–339. 10.1016/s1535-6108(04)00081-9 15093540

[B5] Bassat E. Mutlak Y. E. Genzelinakh A. Shadrin I. Y. Umansky K. B. Yifa O. (2017). The extracellular matrix protein agrin promotes heart regeneration in mice. *Nature* 547 179–184. 10.1038/nature22978 28581497 PMC5769930

[B6] Bennet S. J. Oldridge N. B. Eckert G. J. Embree J. L. Browning S. Hou N. (2002). Discriminant properties of commonly used quality of life measures in heart failure. *Qual. Life Res.* 11 349–359. 10.1023/a:1015547713061 12086120

[B7] Bergmann O. Zdunek S. Felker A. Salehpour M. Alkass K. Bernard S. (2015). Dynamics of cell generation and turnover in the human heart. *Cell* 161, 1566–1575. 10.1016/j.cell.2015.05.026 26073943

[B8] Bersell K. Arab S. Haring B. Kühn B. (2009). Neuregulin1/ErbB4 signaling induces cardiomyocyte proliferation and repair of heart injury. *Cell* 138 257–270. 10.1016/j.cell.2009.04.060 19632177

[B9] Bogush N. Tan L. Naib H. Faizullabhoy E. Calvert J. W. Iismaa S. E. (2020). DUSP5 expression in left ventricular cardiomyocytes of young hearts regulates thyroid hormone (T3)-induced proliferative ERK1/2 signaling. *Sci. Rep.* 10:21918. 10.1038/s41598-020-78825-x 33318551 PMC7736286

[B10] Botting K. J. Wang K. C. Padhee M. McMillen I. C. Summers-Pearce B. Rattanatray L. (2012). Early origins of heart disease: low birth weight and determinants of cardiomyocyte endowment. *Clin. Exp. Pharmaco.l Physiol.* 39 814–823. 10.1111/j.1440-1681.2011.05649.x 22126336

[B11] Cai F. Luis M. A. F. Lin X. Wang M. Cai L. Cen C. (2019). Anthracycline-induced cardiotoxicity in the chemotherapy treatment of breast cancer: preventive strategies and treatment. *Mol. Clin. Oncol.* 11 15–23. 10.3892/mco.2019.1854 31289672 PMC6535635

[B12] Caspi O. Lesman A. Basevitch Y. Gepstein A. Arbel G. Habib I. H. (2007). Tissue engineering of vascularized cardiac muscle from human embryonic stem cells. *Circ. Res.* 100 263–272. 10.1161/01.RES.0000257776.05673.ff 17218605

[B13] Celton-Morizur S. Merlen G. Couton D. Margall-Ducos G. Desdouets C. (2009). The insulin/Akt pathway controls a specific cell division program that leads to generation of binucleated tetraploid liver cells in rodents. *J. Clin. Invest.* 119 1880–1887. 10.1172/jci38677 19603546 PMC2701880

[B14] Chaudhry H. W. Dashoush N. H. Tang H. Zhang L. Wang X. Wu E. X. (2004). Cyclin A2 mediates cardiomyocyte mitosis in the postmitotic myocardium. *J. Biol. Chem.* 279 35858–33866. 10.1074/jbc.M404975200 15159393

[B15] Chen C. S. Mrksich M. Huang S. Whitesides G. M. Ingber D. E. (1997). Geometric control of cell life and death. *Science* 276 1425–1428. 10.1126/science.276.5317.1425 9162012

[B16] Chen X. Wilson R. M. Kubo H. Berretta R. M. Harris D. M. Zhang X. (2007). Adolescent feline heart contains a population of small, proliferative ventricular myocytes with immature physiological properties. *Circ. Res.* 100 536–544. 10.1161/01.RES.0000259560.39234.99 17272809

[B17] Chong J. J. Yang X. Don C. W. Minami E. Liu Y. W. Weyers J. J. (2014). Human embryonic-stem-cell-derived cardiomyocytes regenerate non-human primate hearts. *Nature* 510 273–277. 10.1038/nature13233 24776797 PMC4154594

[B18] Chugh A. R. Beache G. M. Loughran J. H. Mewton N. Elmore J. B. Kajstura J. (2012). Administration of cardiac stem cells in patients with ischemic cardiomyopathy: the SCIPIO trial: surgical aspects and interim analysis of myocardial function and viability by magnetic resonance. *Circulation* 126 S54–S64. 10.1161/CIRCULATIONAHA.112.092627 22965994 PMC3448934

[B19] Derks W. Bergmann O. (2020). Polyploidy in cardiomyocytes: roadblock to heart regeneration? *Circ. Res.* 126 552–565. 10.1161/CIRCRESAHA.119.315408 32078450

[B20] Dhahri W. Dussault S. Légaré É Rivard F. Desjarlais M. Mathieu R. (2020). Reduced expression of microRNA-130a promotes endothelial cell senescence and age-dependent impairment of neovascularization. *Aging (Albany NY)* 12 10180–10193. 10.18632/aging.103340 32457253 PMC7346016

[B21] Dong S. Cheng Y. Yang J. Li J. Liu X. Wang X. (2009). MicroRNA expression signature and the role of microRNA-21 in the early phase of acute myocardial infarction. *J. Biol. Chem.* 284 29514–29525. 10.1074/jbc.M109.027896 19706597 PMC2785585

[B22] Dracup K. Walden J. A. Stevenson L. W. Brecht M. L. (1992). Quality of life in patients with advanced heart failure. *J. Heart Lung Transplant.* 11 273–279. 1576133

[B23] D’Uva G. Aharonov A. Lauriola M. Kain D. Yahalom-Ronen Y. Carvalho S. (2015). ERBB2 triggers mammalian heart regeneration by promoting cardiomyocyte dedifferentiation and proliferation. *Nat. Cell Biol.* 17 627–638. 10.1038/ncb3149 25848746

[B24] Ellison G. M. Vicinanza C. Smith A. J. Aquila I. Leone A. Waring C. D. (2013). Adult c-kit(pos) cardiac stem cells are necessary and sufficient for functional cardiac regeneration and repair. *Cell* 154 827–842. 10.1016/j.cell.2013.07.039 23953114

[B25] Engel F. B. Hsieh P. C. Lee R. T. Keating M. T. (2006). FGF1/p38 MAP kinase inhibitor therapy induces cardiomyocyte mitosis, reduces scarring, and rescues function after myocardial infarction. *Proc. Natl. Acad. Sci. U S A.* 103 15546–15551. 10.1073/pnas.0607382103 17032753 PMC1622860

[B26] Engel F. B. Schebesta M. Duong M. T. Lu G. Ren S. Madwed J. B. (2005). p38 MAP kinase inhibition enables proliferation of adult mammalian cardiomyocytes. *Genes Dev.* 19 1175–1187. 10.1101/gad.1306705 15870258 PMC1132004

[B27] Epelman S. Liu P. P. Mann D. L. (2015). Role of innate and adaptive immune mechanisms in cardiac injury and repair. *Nat. Rev. Immunol.* 15 117–129. 10.1038/nri3800 25614321 PMC4669103

[B28] Fan D. Takawale A. Lee J. Kassiri Z. (2012). Cardiac fibroblasts, fibrosis and extracellular matrix remodeling in heart disease. *Fibrogenesis Tissue Repair.* 5:15. 10.1186/1755-1536-5-15 22943504 PMC3464725

[B29] Fernandes S. Naumova A. V. Zhu W. Z. Laflamme M. A. Gold J. Murry C. E. (2010). Human embryonic stem cell-derived cardiomyocytes engraft but do not alter cardiac remodeling after chronic infarction in rats. *J. Mol. Cell Cardiol.* 49 941–949. 10.1016/j.yjmcc.2010.09.008 20854826 PMC2992844

[B30] Forhead A. J. Fowden A. L. (2014). Thyroid hormones in fetal growth and prepartum maturation. *J. Endocrinol.* 221, R87–R103. 10.1530/JOE-14-0025 24648121

[B31] Gabisonia K. Prosdocimo G. Aquaro G. D. Carlucci L. Zentilin L. Secco I. (2019). MicroRNA therapy stimulates uncontrolled cardiac repair after myocardial infarction in pigs. *Nature* 569 418–422. 10.1038/s41586-019-1191-6 31068698 PMC6768803

[B32] Gao F. Kataoka M. Liu N. Liang T. Huang Z. P. Gu F. (2019). Therapeutic role of miR-19a/19b in cardiac regeneration and protection from myocardial infarction. *Nat. Commun.* 10:1802. 10.1038/s41467-019-09530-1 30996254 PMC6470165

[B33] Gao Y. Pu J. (2021). Differentiation and application of human pluripotent stem cells derived cardiovascular cells for treatment of heart diseases: promises and challenges. *Front. Cell Dev. Biol.* 9:658088. 10.3389/fcell.2021.658088 34055788 PMC8149736

[B34] Gaudron P. Eilles C. Kugler I. Ertl G. (1993). Progressive left ventricular dysfunction and remodeling after myocardial infarction. Potential mechanisms and early predictors. *Circulation* 87 755–763. 10.1161/01.cir.87.3.755 8443896

[B35] Haddad F. Qin A. X. Bodell P. W. Jiang W. Giger J. M. Baldwin K. M. (2008). Intergenic transcription and developmental regulation of cardiac myosin heavy chain genes. *Am. J. Physiol. Heart Circ. Physiol.* 294 H29–H40. 10.1152/ajpheart.01125.2007 17982008

[B36] Heallen T. Zhang M. Wang J. Bonilla-Claudio M. Klysik E. Johnson R. L. (2011). Hippo pathway inhibits Wnt signaling to restrain cardiomyocyte proliferation and heart size. *Science* 332 458–461. 10.1126/science.1199010 21512031 PMC3133743

[B37] Hirai M. Chen J. Evans S. M. (2016). Tissue-Specific cell cycle indicator reveals unexpected findings for cardiac myocyte proliferation. *Circ. Res.* 118 20–28. 10.1161/CIRCRESAHA.115.307697 26472817 PMC5533092

[B38] Hirose K. Payumo A. Y. Cutie S. Hoang A. Zhang H. Guyot R. (2019). Evidence for hormonal control of heart regenerative capacity during endothermy acquisition. *Science* 364 184–188. 10.1126/science.aar2038 30846611 PMC6541389

[B39] Hobbs F. D. Kenkre J. E. Roalfe A. K. Davis R. C. Hare R. Davies M. K. (2002). Impact of heart failure and left ventricular systolic dysfunction on quality of life: a cross-sectional study comparing common chronic cardiac and medical disorders and a representative adult population. *Eur. Heart J.* 23 1867–1876. 10.1053/euhj.2002.3255 12445536

[B40] Ieda M. Fu J. D. Delgado-Olguin P. Vedantham V. Hayashi Y. Bruneau B. G. (2010). Direct reprogramming of fibroblasts into functional cardiomyocytes by defined factors. *Cell* 142 375–386. 10.1016/j.cell.2010.07.002 20691899 PMC2919844

[B41] Iismaa S. E. Li M. Kesteven S. Wu J. Chan A. Y. Holman S. R. (2018). Cardiac hypertrophy limits infarct expansion after myocardial infarction in mice. *Sci. Rep.* 8:6114. 10.1038/s41598-018-24525-6 29666426 PMC5904135

[B42] Ikenishi A. Okayama H. Iwamoto N. Yoshitome S. Tane S. Nakamura K. (2012). Cell cycle regulation in mouse heart during embryonic and postnatal stages. *Dev. Growth Differ.* 54 731–738. 10.1111/j.1440-169X.2012.01373.x 22957921

[B43] Ingason A. B. Goldstone A. B. Paulsen M. J. Thakore A. D. Truong V. N. Edwards B. B. (2018). Angiogenesis precedes cardiomyocyte migration in regenerating mammalian hearts. *J. Thorac. Cardiovasc. Surg.* 155 1118–1127.e1. 10.1016/j.jtcvs.2017.08.127 29452461 PMC5985151

[B44] Jia J. Zhang W. Wang B. Trinko R. Jiang J. (2003). The Drosophila Ste20 family kinase dMST functions as a tumor suppressor by restricting cell proliferation and promoting apoptosis. *Genes Dev.* 17 2514–2519. 10.1101/gad.1134003 14561774 PMC218145

[B45] Jones N. R. Roalfe A. K. Adoki I. Hobbs F. D. R. Taylor C. J. (2019). Survival of patients with chronic heart failure in the community: a systematic review and meta-analysis. *Eur. J. Heart Fail.* 21 1306–1325. 10.1002/ejhf.1594 31523902 PMC6919428

[B46] Kaiser R. A. Bueno O. F. Lips D. J. Doevendans P. A. Jones F. Kimball T. F. (2004). Targeted inhibition of p38 mitogen-activated protein kinase antagonizes cardiac injury and cell death following ischemia-reperfusion in vivo. *J. Biol. Chem.* 279 15524–15530. 10.1074/jbc.M313717200 14749328

[B47] Kanisicak O. Vagnozzi R. J. Molkentin J. D. (2017). Identity crisis for regenerative cardiac cKit+ cells. *Circ. Res.* 121 1130–1132. 10.1161/CIRCRESAHA.117.311921 29074532 PMC5687275

[B48] Krenz M. Robbins J. (2004). Impact of beta-myosin heavy chain expression on cardiac function during stress. *J. Am. Coll. Cardiol.* 44 2390–2397. 10.1016/j.jacc.2004.09.044 15607403

[B49] Kriss J. P. Revesz L. (1962). The distribution and fate of bromodeoxyuridine and bromodeoxycytidine in the mouse and rat. *Cancer Res.* 22 254–265. 14459709

[B50] Kubin T. Pöling J. Kostin S. Gajawada P. Hein S. Rees W. (2011). Oncostatin M is a major mediator of cardiomyocyte dedifferentiation and remodeling. *Cell Stem Cell* 9 420–432. 10.1016/j.stem.2011.08.013 22056139

[B51] Kühn B. del Monte F. Hajjar R. J. Chang Y. S. Lebeche D. Arab S. (2007). Periostin induces proliferation of differentiated cardiomyocytes and promotes cardiac repair. *Nat. Med.* 13 962–969. 10.1038/nm1619 17632525

[B52] Laflamme M. A. Murry C. E. (2011). Heart regeneration. *Nature* 473 326–335. 10.1038/nature10147 21593865 PMC4091722

[B53] Lähteenvuo J. Rosenzweig A. (2012). Effects of aging on angiogenesis. *Circ. Res.* 110 1252–1264. 10.1161/CIRCRESAHA.111.246116 22539758 PMC4101916

[B54] Leach J. P. Heallen T. Zhang M. Rahmani M. Morikawa Y. Hill M. C. (2017). Hippo pathway deficiency reverses systolic heart failure after infarction. *Nature* 550 260–264. 10.1038/nature24045 28976966 PMC5729743

[B55] Li F. Wang X. Capasso J. M. Gerdes A. M. (1996). Rapid transition of cardiac myocytes from hyperplasia to hypertrophy during postnatal development. *J. Mol. Cell Cardiol.* 28 1737–1746. 10.1006/jmcc.1996.0163 8877783

[B56] Li M. Iismaa S. E. Naqvi N. Nicks A. Husain A. Graham R. M. (2014). Thyroid hormone action in postnatal heart development. *Stem Cell Res.* 13 582–591. 10.1016/j.scr.2014.07.001 25087894

[B57] Lin Z. von Gise A. Zhou P. Gu F. Ma Q. Jiang J. (2014). Cardiac-specific YAP activation improves cardiac function and survival in an experimental murine MI model. *Circ. Res.* 115 354–363. 10.1161/CIRCRESAHA.115.303632 24833660 PMC4104149

[B58] Lin Z. Zhou P. von Gise A. Gu F. Ma Q. Chen J. (2015). Pi3kcb links Hippo-YAP and PI3K-AKT signaling pathways to promote cardiomyocyte proliferation and survival. *Circ. Res.* 116 35–45. 10.1161/CIRCRESAHA.115.304457 25249570 PMC4282610

[B59] Lindsey M. L. Bolli R. Canty J. M. Jr. Du X. J. Frangogiannis N. G. Frantz S. (2018). Guidelines for experimental models of myocardial ischemia and infarction. *Am. J. Physiol. Heart Circ. Physiol.* 314 H812–H838. 10.1152/ajpheart.00335.2017 29351451 PMC5966768

[B60] Liu S. Li K. Wagner Florencio L. Tang L. Heallen T. R. Leach J. P. (2021). Gene therapy knockdown of Hippo signaling induces cardiomyocyte renewal in pigs after myocardial infarction. *Sci. Transl. Med.* 13:eabd6892. 10.1126/scitranslmed.abd6892 34193613 PMC9476348

[B61] Liu X. Gu X. Li Z. Li X. Li H. Chang J. (2006). Neuregulin-1/erbB-activation improves cardiac function and survival in models of ischemic, dilated, and viral cardiomyopathy. *J. Am. Coll. Cardiol.* 48 1438–1447. 10.1016/j.jacc.2006.05.057 17010808

[B62] Mahmoud A. I. Kocabas F. Muralidhar S. A. Kimura W. Koura A. S. Thet S. (2013). Meis1 regulates postnatal cardiomyocyte cell cycle arrest. *Nature* 497 249–253. 10.1038/nature12054 23594737 PMC4159712

[B63] Marín-García J. (2010). Thyroid hormone and myocardial mitochondrial biogenesis. *Vascul. Pharmacol.* 52 120–130. 10.1016/j.vph.2009.10.008 19857604

[B64] Marín-Juez R. Marass M. Gauvrit S. Rossi A. Lai S. L. Materna S. C. (2016). Fast revascularization of the injured area is essential to support zebrafish heart regeneration. *Proc. Natl. Acad. Sci. U S A.* 113 11237–11242. 10.1073/pnas.1605431113 27647901 PMC5056108

[B65] McDermott K. Elixhauser A. Sun R. (2017). *Trends in Hospital Inpatient Stays in the United Sates, 2005-2014 HCUP.* Rockville, MD: Agency for Healthcare Research and Quality.

[B66] Mewton N. Croisille P. Gahide G. Rioufol G. Bonnefoy E. Sanchez I. (2010). Effect of cyclosporine on left ventricular remodeling after reperfused myocardial infarction. *J. Am. Coll. Cardiol.* 55 1200–1205. 10.1016/j.jacc.2009.10.052 20298926

[B67] Miyaoka Y. Ebato K. Kato H. Arakawa S. Shimizu S. Miyajima A. (2012). Hypertrophy and unconventional cell division of hepatocytes underlie liver regeneration. *Curr. Biol.* 22 1166–1175. 10.1016/j.cub.2012.05.016 22658593

[B68] Mollova M. Bersell K. Walsh S. Savla J. Das L. T. Park S. Y. (2013). Cardiomyocyte proliferation contributes to heart growth in young humans. *Proc. Natl. Acad. Sci. U S A.* 110 1446–1451. 10.1073/pnas.1214608110 23302686 PMC3557060

[B69] Monroe T. O. Hill M. C. Morikawa Y. Leach J. P. Heallen T. Cao S. (2019). YAP partially reprograms chromatin accessibility to directly induce adult cardiogenesis in vivo. *Dev. Cell* 48 765–779.e7. 10.1016/j.devcel.2019.01.017 30773489 PMC6435425

[B70] Murray T. V. Smyrnias I. Schnelle M. Mistry R. K. Zhang M. Beretta M. (2015). Redox regulation of cardiomyocyte cell cycling via an ERK1/2 and c-Myc-dependent activation of cyclin D2 transcription. *J. Mol. Cell Cardiol.* 79 54–68. 10.1016/j.yjmcc.2014.10.017 25450615 PMC4312357

[B71] Nakada Y. Canseco D. C. Thet S. Abdisalaam S. Asaithamby A. Santos C. X. (2017). Hypoxia induces heart regeneration in adult mice. *Nature* 541 222–227. 10.1038/nature20173 27798600

[B72] Naqvi N. Li M. Calvert J. W. Tejada T. Lambert J. P. Wu J. (2014). A proliferative burst during preadolescence establishes the final cardiomyocyte number. *Cell* 157 795–807. 10.1016/j.cell.2014.03.035 24813607 PMC4078902

[B73] Naqvi N. Singh R. Iismaa S. E. Li M. Calvert J. W. Martin D. I. (2015). Cardiomyocytes replicate and their numbers increase in young hearts. *Cell* 163 783–784. 10.1016/j.cell.2015.10.038 26544928 PMC5875184

[B74] Orlic D. Kajstura J. Chimenti S. Jakoniuk I. Anderson S. M. Li B. (2001). Bone marrow cells regenerate infarcted myocardium. *Nature* 410 701–705. 10.1038/35070587 11287958

[B75] Paige S. L. Thomas S. Stoick-Cooper C. L. Wang H. Maves L. Sandstrom R. (2012). A temporal chromatin signature in human embryonic stem cells identifies regulators of cardiac development. *Cell* 151 221–232. 10.1016/j.cell.2012.08.027 22981225 PMC3462257

[B76] Palmen M. Daemen M. J. De Windt L. J. Willems J. Dassen W. R. Heeneman S. (2004). Fibroblast growth factor-1 improves cardiac functional recovery and enhances cell survival after ischemia and reperfusion: a fibroblast growth factor receptor, protein kinase C, and tyrosine kinase-dependent mechanism. *J. Am. Coll. Cardiol.* 44 1113–1123. 10.1016/j.jacc.2004.05.067 15337227

[B77] Pantos C. Xinaris C. Mourouzis I. Malliopoulou V. Kardami E. Cokkinos D. V. (2007). Thyroid hormone changes cardiomyocyte shape and geometry via ERK signaling pathway: potential therapeutic implications in reversing cardiac remodeling? *Mol. Cell Biochem.* 297 65–72. 10.1007/s11010-006-9323-3 17024559

[B78] Pasumarthi K. B. Nakajima H. Nakajima H. O. Soonpaa M. H. Field L. J. (2005). Targeted expression of cyclin D2 results in cardiomyocyte DNA synthesis and infarct regression in transgenic mice. *Circ. Res.* 96 110–118. 10.1161/01.RES.0000152326.91223.4F 15576649

[B79] Patterson M. Barske L. Van Handel B. Rau C. D. Gan P. Sharma A. (2017). Frequency of mononuclear diploid cardiomyocytes underlies natural variation in heart regeneration. *Nat. Genet.* 49 1346–1353. 10.1038/ng.3929 28783163 PMC5736145

[B80] Piot C. Croisille P. Staat P. Thibault H. Rioufol G. Mewton N. (2008). Effect of cyclosporine on reperfusion injury in acute myocardial infarction. *N. Engl. J. Med.* 359 473–481. 10.1056/NEJMoa071142 18669426

[B81] Polizzotti B. D. Ganapathy B. Walsh S. Choudhury S. Ammanamanchi N. Bennett D. G. (2015). Neuregulin stimulation of cardiomyocyte regeneration in mice and human myocardium reveals a therapeutic window. *Sci. Transl. Med.* 7:281ra45. 10.1126/scitranslmed.aaa5171 25834111 PMC5360874

[B82] Porrello E. R. Mahmoud A. I. Simpson E. Hill J. A. Richardson J. A. Olson E. N. (2011). Transient regenerative potential of the neonatal mouse heart. *Science* 331 1078–1080. 10.1126/science.1200708 21350179 PMC3099478

[B83] Pride Y. B. Giuseffi J. L. Mohanavelu S. Harrigan C. J. Manning W. J. Gibson C. M. (2010). Relation between infarct size in ST-segment elevation myocardial infarction treated successfully by percutaneous coronary intervention and left ventricular ejection fraction three months after the infarct. *Am. J. Cardiol.* 106 635–640. 10.1016/j.amjcard.2010.04.012 20723637

[B84] Puente B. N. Kimura W. Muralidhar S. A. Moon J. Amatruda J. F. Phelps K. L. (2014). The oxygen-rich postnatal environment induces cardiomyocyte cell-cycle arrest through DNA damage response. *Cell* 157 565–579. 10.1016/j.cell.2014.03.032 24766806 PMC4104514

[B85] Reuter S. Soonpaa M. H. Firulli A. B. Chang A. N. Field L. J. (2014). Recombinant neuregulin 1 does not activate cardiomyocyte DNA synthesis in normal or infarcted adult mice. *PLoS One* 9:e115871. 10.1371/journal.pone.0115871 25545368 PMC4278834

[B86] Richardson W. J. Clarke S. A. Quinn T. A. Holme S. J. W. (2015). Physiological implications of myocardial scar structure. *Compr. Physiol.* 5 1877–1909. 10.1002/cphy.c140067 26426470 PMC4727398

[B87] Roes S. D. Kelle S. Kaandorp T. A. Kokocinski T. Poldermans D. Lamb H. J. (2007). Comparison of myocardial infarct size assessed with contrast-enhanced magnetic resonance imaging and left ventricular function and volumes to predict mortality in patients with healed myocardial infarction. *Am. J. Cardiol.* 100 930–936. 10.1016/j.amjcard.2007.04.029 17826372

[B88] Ruoslahti E. (1996). RGD and other recognition sequences for integrins. *Annu. Rev. Cell Dev. Biol.* 12 697–715. 10.1146/annurev.cellbio.12.1.697 8970741

[B89] Sadahiro T. Yamanaka S. Ieda M. (2015). Direct cardiac reprogramming: progress and challenges in basic biology and clinical applications. *Circ. Res.* 116 1378–1391. 10.1161/CIRCRESAHA.116.305374 25858064

[B90] Shi Y. Moon M. Dawood S. McManus B. Liu P. P. (2010). Mechanisms and management of doxorubicin cardiotoxicity. *Herz* 36 296–305. 10.1007/s00059-011-3470-3 21656050

[B91] Shimazaki M. Nakamura K. Kii I. Kashima T. Amizuka N. Li M. (2008). Periostin is essential for cardiac healing after acute myocardial infarction. *J. Exp. Med.* 205 295–303. 10.1084/jem.20071297 18208976 PMC2271007

[B92] Sidney S. Sorel M. E. Quesenberry C. P. Jaffe M. G. Solomon M. D. Nguyen-Huynh M. N. (2018). Comparative trends in heart disease, stroke, and all-cause mortality in the united states and a large integrated healthcare delivery system. *Am. J. Med.* 131 829–836.e1. 10.1016/j.amjmed.2018.02.014 29625083 PMC6005733

[B93] Song K. Nam Y. J. Luo X. Qi X. Tan W. Huang G. N. (2012). Heart repair by reprogramming non-myocytes with cardiac transcription factors. *Nature* 485 599–604. 10.1038/nature11139 22660318 PMC3367390

[B94] Soonpaa M. H. Kim K. K. Pajak L. Franklin M. Field L. J. (1996). Cardiomyocyte DNA synthesis and binucleation during murine development. *Am. J. Physiol.* 271 H2183–H2189. 10.1152/ajpheart.1996.271.5.H2183 8945939

[B95] Soonpaa M. H. Koh G. Y. Pajak L. Jing S. Wang H. Franklin M. T. (1997). Cyclin D1 overexpression promotes cardiomyocyte DNA synthesis and multinucleation in transgenic mice. *J. Clin. Invest.* 99 2644–2654. 10.1172/JCI119453 9169494 PMC508110

[B96] Soonpaa M. H. Zebrowski D. C. Platt C. Rosenzweig A. Engel F. B. Field L. J. (2015). Cardiomyocyte cell-cycle activity during preadolescence. *Cell* 163 781–792. 10.1016/j.cell.2015.10.037 26544927

[B97] Sutton M. G. Sharpe N. (2000). Left ventricular remodeling after myocardial infarction: pathophysiology and therapy. *Circulation* 101 2981–2988. 10.1161/01.cir.101.25.2981 10869273

[B98] Tan L. Bogush N. Naib H. Perry J. Calvert J. W. Martin D. I. K. (2019). Redox activation of JNK2α2 mediates thyroid hormone-stimulated proliferation of neonatal murine cardiomyocytes. *Sci. Rep.* 9:17731. 10.1038/s41598-019-53705-1 31776360 PMC6881338

[B99] Tan L. Bogush N. Naqvi E. Calvert J. W. Graham R. M. Taylor W. R. (2021). Thyroid hormone plus dual-specificity phosphatase-5 siRNA increases the number of cardiac muscle cells and improves left ventricular contractile function in chronic doxorubicin-injured hearts. *Theranostics* 11 4790–4808. 10.7150/thno.57456 33754028 PMC7978295

[B100] Taylor C. J. Ryan R. Nichols L. Gale N. Hobbs F. R. Marshall T. (2017). Survival following a diagnosis of heart failure in primary care. *Fam. Pract.* 34 161–168. 10.1093/fampra/cmw145 28137979 PMC6192063

[B101] Tejada T. Tan L. Torres R. A. Calvert J. W. Lambert J. P. Zaidi M. (2016). IGF-1 degradation by mouse mast cell protease 4 promotes cell death and adverse cardiac remodeling days after a myocardial infarction. *Proc. Natl. Acad. Sci. U S A.* 113 6949–6954. 10.1073/pnas.1603127113 27274047 PMC4922143

[B102] Turrens J. F. (2003). Mitochondrial formation of reactive oxygen species. *J. Physiol.* 552 335–344. 10.1113/jphysiol.2003.049478 14561818 PMC2343396

[B103] Ungvari Z. Tarantini S. Donato A. J. Galvan V. Csiszar A. (2018). Mechanisms of vascular aging. *Circ. Res.* 123 849–867. 10.1161/CIRCRESAHA.118.311378 30355080 PMC6248882

[B104] Urbich M. Globe G. Pantiri K. Heisen M. Bennison C. Wirtz H. S. (2020). A systematic review of medical costs associated with heart failure in the USA (2014-2020). *Pharmacoeconomics* 38 1219–1236. 10.1007/s40273-020-00952-0 32812149 PMC7546989

[B105] Vagnozzi R. J. Maillet M. Sargent M. A. Khalil H. Johansen A. K. Z. Schwanekamp J. A. (2020). An acute immune response underlies the benefit of cardiac stem cell therapy. *Nature* 577 405–409. 10.1038/s41586-019-1802-2 31775156 PMC6962570

[B106] van Berlo J. H. Kanisicak O. Maillet M. Vagnozzi R. J. Karch J. Lin S. C. (2014). c-kit+ cells minimally contribute cardiomyocytes to the heart. *Nature* 509 337–341. 10.1038/nature13309 24805242 PMC4127035

[B107] Ventura-Clapier R. Garnier A. Veksler V. (2004). Energy metabolism in heart failure. *J. Physiol.* 555 1–13. 10.1113/jphysiol.2003.055095 14660709 PMC1664831

[B108] von Gise A. Lin Z. Schlegelmilch K. Honor L. B. Pan G. M. Buck J. N. (2012). YAP1, the nuclear target of Hippo signaling, stimulates heart growth through cardiomyocyte proliferation but not hypertrophy. *Proc. Natl. Acad. Sci. U S A.* 109 2394–2399. 10.1073/pnas.1116136109 22308401 PMC3289361

[B109] Wamstad J. A. Alexander J. M. Truty R. M. Shrikumar A. Li F. Eilertson K. E. (2012). Dynamic and coordinated epigenetic regulation of developmental transitions in the cardiac lineage. *Cell* 151 206–220. 10.1016/j.cell.2012.07.035 22981692 PMC3462286

[B110] Wang J. Karra R. Dickson A. L. Poss K. D. (2013). Fibronectin is deposited by injury-activated epicardial cells and is necessary for zebrafish heart regeneration. *Dev. Biol.* 382 427–435. 10.1016/j.ydbio.2013.08.012 23988577 PMC3852765

[B111] Wang W. E. Li L. Xia X. Fu W. Liao Q. Lan C. (2017). Dedifferentiation, proliferation, and redifferentiation of adult mammalian cardiomyocytes after ischemic injury. *Circulation* 136 834–848. 10.1161/CIRCULATIONAHA.116.024307 28642276 PMC5575972

[B112] Wells R. G. (2008). The role of matrix stiffness in regulating cell behavior. *Hepatology* 7 1394–1400. 10.1002/hep.22193 18307210

[B113] White H. D. Norris R. M. Brown M. A. Brandt P. W. Whitlock R. M. Wild C. J. (1987). Left ventricular end-systolic volume as the major determinant of survival after recovery from myocardial infarction. *Circulation* 76, 44–51. 10.1161/01.cir.76.1.44 3594774

[B114] Woo Y. J. Panlilio C. M. Cheng R. K. Liao G. P. Atluri P. Hsu V. M. (2006). Therapeutic delivery of cyclin A2 induces myocardial regeneration and enhances cardiac function in ischemic heart failure. *Circulation* 114 I206–I1213. 10.1161/CIRCULATIONAHA.105.000455 16820573

[B115] Wu M. Y. Yiang G. T. Liao W. T. Tsai A. P. Cheng Y. L. Cheng P. W. (2018). Current mechanistic concepts in ischemia and reperfusion injury. *Cell Physiol. Biochem.* 46 1650–1667. 10.1159/000489241 29694958

[B116] Xin M. Kim Y. Sutherland L. B. Qi X. McAnally J. Schwartz R. J. (2011). Regulation of insulin-like growth factor signaling by Yap governs cardiomyocyte proliferation and embryonic heart size. *Sci. Signal.* 4:ra70. 10.1126/scisignal.2002278 22028467 PMC3440872

[B117] Yamakawa H. Ieda M. (2021). Cardiac regeneration by direct reprogramming in this decade and beyond. *Inflamm. Regen.* 41:20. 10.1186/s41232-021-00168-5 34193320 PMC8247073

[B118] Yang F. Liu Y. H. Yang X. P. Xu J. Kapke A. Carretero O. A. (2002). Myocardial infarction and cardiac remodelling in mice. *Exp. Physiol.* 87 547–555. 10.1113/eph8702385 12481929

[B119] Yang Y. Cheng H. W. Qiu Y. Dupee D. Noonan M. Lin Y. D. (2015). MicroRNA-34a plays a key role in cardiac repair and regeneration following myocardial infarction. *Circ. Res.* 117 450–459. 10.1161/CIRCRESAHA.117.305962 26082557 PMC4769861

[B120] Yellon D. M. Hausenloy D. J. (2007). Myocardial reperfusion injury. *N. Engl. J. Med.* 357 1121–1135. 10.1056/NEJMra071667 17855673

[B121] Zwetsloot P. P. Végh A. M. Jansen of Lorkeers S. J. van Hout G. P. Currie G. L. Sena E. S. (2016). Cardiac stem cell treatment in myocardial infarction: a systematic review and meta-analysis of preclinical studies. *Circ. Res.* 118 1223–1232. 10.1161/CIRCRESAHA.115.307676 26888636

